# The Role of Lanthanum Stearate on Strain-Induced Crystallization and the Mechanical Properties of Whole Field Latex Rubber

**DOI:** 10.3390/polym16020276

**Published:** 2024-01-19

**Authors:** Changjin Yang, Yuhang Luo, Zechun Li, Chuanyu Wei, Shuangquan Liao

**Affiliations:** 1 School of Materials Science and Engineering, Hainan University, Haikou 570228, China; yj87211@163.com; 2 Key Laboratory of Advanced Materials of Tropical Island Resources, Ministry of Education, Hainan University, Haikou 570228, China; leoyuhang18@icloud.com (Y.L.); 17886719350@163.com (Z.L.); 13256357281@163.com (C.W.)

**Keywords:** lanthanum stearate, NR, SCR-WF, mechanical property, strain-induced crystallization

## Abstract

Natural rubber (NR) is extensively utilized in numerous industries, such as aerospace, military, and transportation, because of its exceptional elasticity and all-around mechanical qualities. However, commercial NR made using various techniques typically has distinct mechanical characteristics. For instance, whole field latex rubber (SCR-WF) cured with accelerator 2-Mercaptobenzothiazole exhibits poor mechanical properties. This work attempts to enhance the mechanical property of SCR-WF via the addition of lanthanum stearate (LaSt). The influence of LaSt on strain-induced crystallization (SIC) and the mechanical properties of SCR-WF were investigated. The results of crosslinking density measured by the equilibrium swelling method demonstrate that the presence of LaSt significantly increases the crosslinking density of SCR-WF with lower loading of LaSt. The results of the mechanical properties show that the introduction of LaSt can enhance the tensile strength and fracture toughness of SCR-WF. To reveal the mechanism of LaSt improving the mechanical properties of SCR-WF, synchrotron radiation wide-angle X-ray diffraction (WAXD) experiments were used to investigate the SIC behaviors of SCR-WF. We found that the LaSt leads to higher crystallinity of SIC for the strain higher than 3.5. The tube model indicates the contribution of LaSt in both crosslinking and topological constraints. This work may provide an instruction for developing SCR-WF with superior mechanical properties.

## 1. Introduction

Natural rubber (NR) is an important commercial raw material for rubber industries, owing to outstanding comprehensive properties, such as higher tensile strength and tear strength, low heat build-up and excellent antifatigue resistance [1,2]. Due to these unique properties, NR has been widely used in airplane tires, bearings of high-speed strains, and other high-performance rubber products [3]. These unique properties are mainly ascribed to strain-induced crystallization (SIC) because of the high stereoregularity [4]. This property was first discovered in 1925 by Katz [5]; a great deal of research endeavors have been dedicated to the investigation of SIC of NR via many techniques, such as infrared spectroscopy [6], nuclear magnetic resonance [7,8], volume change [9,10], birefringence [11], stress relaxation [12], X-ray diffraction [13], and so on. At present, the X-ray diffraction technique is extensively used to study the structure and lattice parameters of the strain-induced crystals in NR vulcanizates [13,14,15].

Recently, it has been reported that rare-earth complexes significantly improve the aging resistance of rubber owing to many unoccupied orbits of rare-earth ions, which exhibits great scavenging effects on free radicals [16,17]. Qiu [18] reported that composites containing rare earth possess higher mechanical and thermo-oxidative aging resistance due to some “instantaneous complex”, which is produced when vulcanized samples are stretched. Xie [19] and co-workers found that the Dy(III) complex had significant effects on capturing free radicals during the aging process. Zheng [20] also indicated that DaGLa could enhance the thermo-oxidative stability of NR. Fang [21] reported that the combination of Ca/Z stabilizers and LaSt showed an obvious improvement of stabilization efficiency for polyvinyl chloride (PVC) in air when the temperature is 180 °C. In our previous study, we found that rare-earth stearate [16,22,23] can prominently improve the hot air aging resistance of epoxidized natural rubber.

Commercial NR made using various techniques typically has distinct mechanical characteristics. SCR-WF is one of the technical graded rubbers of commercial NR. And it is usually obtained by coagulating with acid, flaking, granulating, and drying. By far, the most commonly used acids for commercial NR of SCR-WF are acetic acid, formic acid, and sulfuric acid [24,25]. Usually, NR coagulated with acetic acid cured with 2-Mercaptobenzothiazole shows poorer tensile strength and tear strength than that of microbial coagulated NR [26,27], because rare-earth compounds can improve the mechanical properties of rubber. Meanwhile, LaSt is an industrial product, easy to obtain, and an excellent heat stabilizer, and is widely used in the plastic industry. So, we aimed to use LaSt as a functional modifier to improve the mechanical properties and other comprehensive properties of SCR-WF.

In the present work, we study the role of LaSt on the SIC behavior and mechanical properties of SCR-WF. The network structure of vulcanized NR was investigated. To further study the reinforcement mechanism of LaSt, synchrotron radiation wide-angle X-ray diffraction (WAXD) experiments were used to investigate the strain-induced crystallization (SIC) behaviors of NR without and with LaSt. Some unexpected results were gained, as well as new insights on the relationship of crystallinity and mechanical properties for NR vulcanizates with LaSt. It is expected that the experiment results will provide an instruction for developing whole field latex rubber with superior mechanical properties and help expand the application of whole field latex rubber in rubber products.

## 2. Materials and Methods

### 2.1. Materials

Commercial-grade standard natural rubber (type SCR-WF) was used and manufactured by Yunnan Natural Rubber Industrial Co., Ltd., Kunming, China. Accelerator 2-mercaptobenzothiazole (M) was purchased from Aladdin, Shanghai, China. Activator zinc oxide (ZnO) and stearic acid were purchased from Shanghai Macklin Biochem. Co., Ltd., Shanghai, China. Sulfur and LaSt were of commercial grades and used as supplied.

### 2.2. Preparation of Samples

The base formulation of NR compounds was as follows (phr): NR, 100; sulfur, 3.5; accelerator M, 0.5; zinc oxide, 6; stearic acid, 0.5; LaSt, 0~2.

Mixing was carried out by using a two-roll mill at room temperature. The total mixing time was 10 min in one mixing cycle. The rubber compounds were left at room temperature for 8 h before testing. Vulcanization was carried out in an electrically heated hydraulic press at 140 °C using the optimum cure time (T_90_) previously determined with a vulcanizer.

### 2.3. Methods

Cure characteristics of NR compound were calculated using Moving Die Rheometer (M-3000AU from Gotech Testing Machines Inc., Taiwan, China) according to ASTM D2084-01 standard [28] tested at 140 °C. The cure rate index was calculated from Equation (1) as follows:

(1) $$CRI=\frac{1}{T_{90}-T_{S1}}$$

where *T*_90_ is cure time and *T*_S1_ is scorch time.

The mechanical properties of the NR vulcanizates were carried out using universal material testing machine GOTECH AI-3000 (Gotech Testing Machines Inc., Taiwan, China) according to the standard method (ASTM D412-06 [29]). The rate of testing speed was 500 mm/min.

Fracture tests were conducted using the classical single edge notch test on the GOTECH AI-3000 testing machine with an extension rate of 6 mm/min at room temperature. The fracture toughness (G) was calculated from Equation (2) as follows [30]:

(2) $$G=\frac{6Wc}{\sqrt{\lambda_{c}}}$$

where *c* is the length of the notch, *λ_c_* is the strain at break of sample, *W* is the strain energy density calculated by integration of the stress–strain curve of un-notched sample until *λ*_c−1_.

Crosslinking density of NR vulcanizates was measured by equilibrium swelling method. The equilibrium swelling measurement was carried our at room temperature in toluene for 7 days. The values of crosslink density of NR are calculated from Equation (3) as follows [31]:

(3) $$\gamma =\frac{-[\ln\left(1-V_{r}\right)+V_{r}+\chi V_{r}^{2}]}{V_{s}\times \left(V_{r}^{1/3}-V_{r}/2\right)}$$

where γ is the number of active network chain segments per unit of volume (crosslinking density), *V*_r_ is the volume fraction of rubber in the swollen network, *V_s_* is the molar volume of the solvent (105.7 cm^3^/mol for toluene), and *χ* is the Flory–Huggins polymer–solvent interaction term (0.393 for NR/toluene).

The Synchrotron WAXD experiments were carried out at room temperature (IUS beam-line, Incoatec, Geesthacht, Germany). The wavelength of X-ray was 0.154 nm. The deformation speed of NR samples was set up as 3 mm/min and kept unchanged for each test. The distance between the NR sample and the detector was 47.1 mm. WAXD patterns were recorded every 60 s with the Dectris Pilatus 300 K detector system (internal strain: 0.15). The WAXD patterns were background-corrected and processed using Fit2D (V18.002) software (the product of European Synchrotron Radiation Facility (ESRF)) for further analysis.

The orientation of the amorphous phase <$P_{2}^{am}$> was calculated by from Equation (4) as follows [32,33,34]:

(4) $$<P_{2}^{am}>=\frac{\int I_{am}(\cos\varphi )P_{2}\left(cos\varphi \right)d(cos\varphi )}{\int I_{am}(\cos\varphi )d(\cos\varphi )}=-\frac{2B}{15A+5B}$$

where $I_{am}$ is the azimuthal scan of the amorphous intensity. For each elongation ratio, the integrated intensity was fitted. *A* and *B* parameters were extracted, then the value of <$P_{2}^{am}$> was calculated.

## 3. Results and Discussion

### 3.1. Cure Characteristics of SCR-WF with Different LaSt Loadings

**Figure 1:**
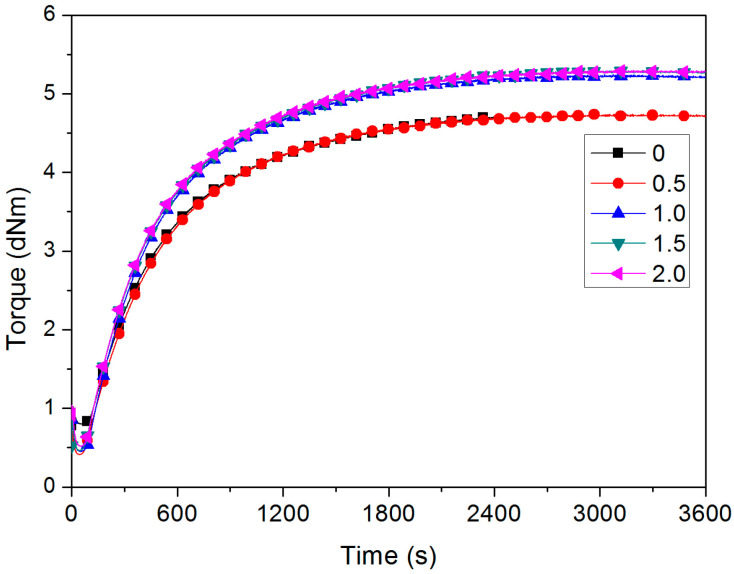
The cure curves of NR compound with different LaSt loadings.

Figure 1 shows the vulcanization curves of SCR-WF with different LaSt loadings. It is clear that the value of maximum torque (M_H_) obviously increases when the LaSt content exceeded 0.5 phr. To obtain more specific information of the cure, some important cure parameters, including the scorch time (T_S1_), cure time (T_90_), maximum torque (M_H_), minimum torque (M_L_), and the difference between minimum and maximum torque (M_H_ − M_L_), were determined from the vulcanization curves, as illustrated in Table 1. It is obvious that the value of T_S1_ of the compound gradually decreases with increasing LaSt loading. But the addition of LaSt has no significant effect on scorch time. It is very important to ensure security during the process of NR products. As the LaSt loading increases, the value of T_90_ and the cure rate index (CRI) also decreases. It is indicated that the addition of LaSt has taken the cure reaction, but it cannot accelerate the vulcanization of SCR-WF. The value of M_H_−M_L_ is proportional to the crosslink density for rubber vulcanizates [35]. It is evident from Table 1 that the value of M_H_−M_L_ increases with increasing LaSt loading. This may be possibly ascribed to the presence of rare-earth La in the NR compound; the La ion may have taken part in the cure reaction to form the more compact crosslinked network structure, because La has the electron shell structure as 5d16s2. The large number of empty orbitals and long atom radius result in strong coordination abilities, which make it easy to form complex. As we all know, NR has 6% non-rubber components, including phospholipids and proteins [36], because La has strong coordination abilities with O, N, and P atoms, which largely exist in the molecular structure of phospholipids and proteins. Therefore, during the vulcanization process, the complexation of La and O, N, P atoms will maybe form more crosslinking points, which results in the increase in M_H_−M_L_.

### 3.2. Crosslink Density Measurements of SCR-WF with Different LaSt Loadings

The crosslink densities of the SCR-WF with various loads of LaSt were calculated by the equilibrium swelling method. And the results of the crosslink densities are shown in Table 2. From Table 2, it can be seen that the crosslink densities of SCR-WF increase with increasing LaSt loading. Crosslink density is usually used to evaluate the mechanical property changes of rubber vulcanizates.

This is consistent with the improvement in M_H_ − M_L_ in the cure characteristics of SCR-WF with the increase in LaSt loading. Namely, the higher the crosslink density is, the higher the modulus will be.

### 3.3. Mechanical Properties of Vulcanized SCR-WF with Different LaSt Loadings

**Figure 2:**
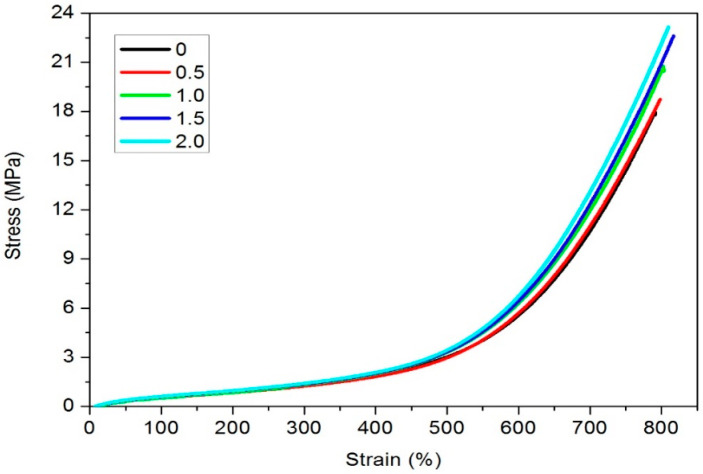
The cure curves of vulcanized SCR-WF with different LaSt loadings.

The stress–strain curves for vulcanized NR with different LaSt loadings are depicted in Figure 2, and the corresponding properties are listed in Table 3. As illustrated in Figure 2, the stress–strain curves of vulcanized SCR-WF with different loadings of LaSt are almost overlapped when the strain is smaller than 4.5, while some difference can be found when the strain exceeds 4.5. From Table 3, it is obvious that the tensile strength of vulcanized SCR-WF increases gradually with increasing LaSt loading. When the content of LaSt reaches 2 phr, the value of tensile strength for SCR-WF increases by 27%, compared with that of NR without LaSt. Furthermore, the moduli 100%, 300%, and 500% of vulcanized SCR-WF increase slightly by enhancing the content of LaSt. However, the elongation at break decreases slightly with increasing loading of LaSt. This observation can also be explained by the higher crosslink density of vulcanized SCR-WF with LaSt.

**Figure 3:**
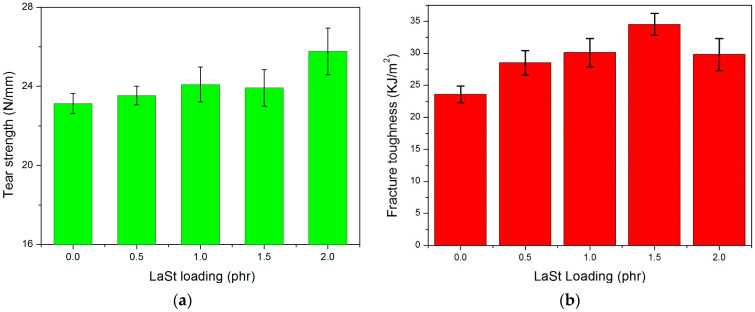
Effect of LaSt loading on the tear strength (**a**) and fracture toughness (**b**) of vulcanized SCR-WF.

Figure 3a illustrates the effect of LaSt loading on the tear strength of vulcanized SCR-WF. It is observed that the tear strength of vulcanized SCR-WF increases gradually with the increase in LaSt contents up to about 1.0 phr of LaSt, after which a drop in tear strength is observed in SCR-WF with 1.5 phr LaSt; then, the tear strength continually increases with a further increase in LaSt loading. However, compared with pure vulcanized SCR-WF, SCR-WF with LaSt shows a higher tear strength. So, the addition of LaSt enhances the tear strength of vulcanized SCR-WF.

Figure 3b illustrates the effect of LaSt loading on the fracture toughness of vulcanized SCR-WF. It can be seen that the fracture toughness of NR vulcanizates increase gradually with increasing LaSt loading up to about 1.5 phr of LaSt, after which a drop of fracture toughness was observed with further increasing LaSt loading. In the notch experiment, the cracks of NR become blunt during large deformation, which effectively reduces the stress concentration at the notch and contributes to energy dissipation [37]. When the amount of LaSt exceeds 1.5 phr, a more rubber entanglement network structure and an effective crosslinked structure will affect the energy dissipation of rubber.

In summary, an appropriate addition of LaSt can improve the crosslinking density and mechanical properties of SCR-WF, which will provide a strong enough crosslinked network structure to resist damage from external forces during the use or service of rubber products.

The introduction of LaSt can enhance the mechanical properties and fracture toughness due to higher crosslink density. Moreover, in previous studies [18], some “instantaneous complex” can be produced when the vulcanized SCR-WF is stretched, because macromolecules are forced to arrange quickly and distance between La atoms and ligand narrows rapidly. The tensile strength, modus, and fracture toughness for SCR-WF increase with the increasing contents of LaSt due to the “instantaneous complex”. To further investigate the reinforcement mechanism of LaSt, the network of vulcanized SCR-WF was analyzed by a tube model, and the SCI behavior of vulcanized SCR-WF with LaSt was performed to elucidate the mechanism of reinforcement in order to explain the superior performance of SCR-WF containing LaSt.

### 3.4. Strain-Induced Crystallization of Vulcanized SCR-WF with 2 phr LaSt and without LaSt

**Figure 4:**
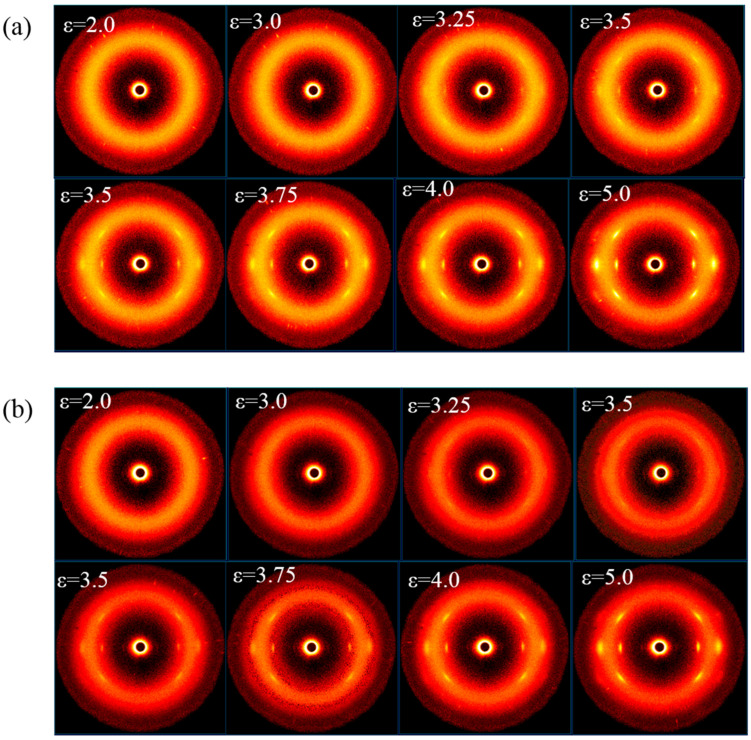
WAXD patterns of (**a**) SCR-WF without LaSt and (**b**) SCR-WF with 2 phr LaSt.

**Figure 5:**
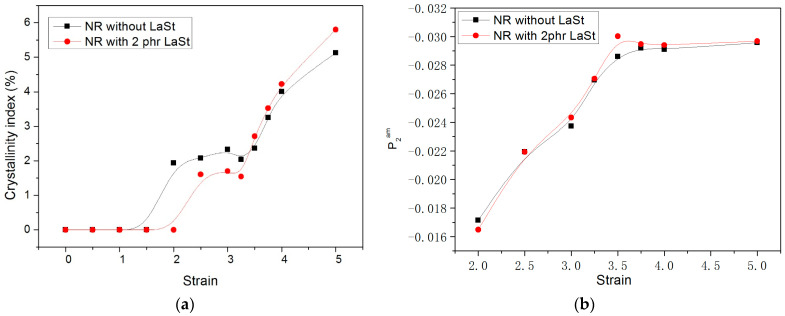
The variation of crystallinity index (**a**) and amorphous orientation parameter <$P_{2}^{am}$> (**b**) of SCR-WF without LaSt and with 2 phr LaSt.

To disclose the influence of LaSt on the SIC behavior of SCR-WF, WAXD experiments are conducted to detect the SIC behavior of SCR-WF without LaSt and 2 phr LaSt. Figure 4 shows typical changes of WAXD patterns of vulcanized SCR-WF without LaSt and with 2 phr LaSt. It is observed that an intense amorphous halo is reserved during the stretching process. At a low strain (ε < 2), the two samples display an amorphous feature. With the increase in the strain, there are some highly oriented crystalline bright arcs appearing, which indicates the onset of SIC of vulcanized SCR-WF. To further quantitatively study the SCI process of vulcanized SCR-WF, the collected WAXD images were background-corrected. They should be transformed to 1D curves by integrating the patterns along the azimuthal direction from 0° to 360°. The resulting profiles were deconvoluted into one amorphous NR peak and three crystal diffractions (corresponding to 200, 201, 120 lattice planes), as illustrated in Figure 5a,b. Then, the crystallinity ($\chi_{c}$) was calculated from diffraction intensity data using Equation (5) [38,39]:

(5) $$\chi_{c}\left(\%\right)=\frac{A_{c}}{A_{C}+A_{a}}\times 100$$

where $A_{C}$ is the sum area below the 200, 201, and 120 crystalline peaks, and $A_{a}$ represents the integrated area of the amorphous peak.

Figure 5a shows the calculated crystallinity index of SCR-WF without LaSt and SCR-WF with 2 phr LaSt. It can be clearly seen that vulcanized SCR-WF without LaSt is the first to crystallize, while the onset strain of SIC of vulcanized SCR-WF with LaSt is around 2.5. It is indicated that the addition of LaSt cannot accelerate the onset strain of SIC of SCR-WF, although the crosslink density of vulcanized SCR-WF with LaSt is higher than pure vulcanized SCR-WF. These observations are consistent with the previous reported results; it is shown that crosslink density cannot change the onset strain of SIC, although the higher-density rubber samples create a large amount of SIC at a larger strain [12]. At the same time, the crystallinity index of SCR-WF without LaSt is lower than that of SCR-WF with LaSt when the strain is lower than 3.5. Only when the strain is larger than 3.5 does the vulcanized SCR-WF with 2 phr LaSt exhibit a higher crystallinity index than SCR-WF without LaSt, which indicates that LaSt can enhance the crystallinity index. Under a large enough deformation, the crystals induced by stretching could fix the oriented molecule and influence the overall orientation of rubber molecular chains [40].

The variation in <$P_{2}^{am}$> as a function of strain is shown in Figure 5b. It is observed that the two curves deviate from linearity and reach a plateau because of the so-called strain regulation effect above the SCI onset [41]. In addition, compared with SCR-WF without LaSt, NR with LaSt has a little higher value of <$P_{2}^{am}$> at the strain lager than 3.25. The trend is the same as the results of the crystallinity index. It can be concluded that LaSt could make NR molecular chain orientation easier when the strain is greater than 3.5. This may be possible because LaSt can react with non-rubber components (proteins and phospholipids) in NR to form some instant complexes under a sufficiently large strain, making it easier to orient the molecular chain.

In order to explain the improved mechanical properties of SCR-WF with LaSt, the influence of LaSt on the network topology of vulcanized SCR-WF is further analyzed by using the tube model theory. There are some studies on using typical tube models to study rubber networks [42,43]. According to this model, the constitutive equation of a uniaxial deformation of an incompressible sample is composed of two contributions, as shown in Equations (6) and (7), based on the tube model [44]:

(6) $$\sigma_{M}=\frac{\sigma }{\sigma -\sigma^{-2}}=G_{c}+G_{e}f(\alpha )$$

(7) $$f\left(\alpha \right)=\frac{2}{\beta }\;\frac{\alpha^{\beta /2}-\alpha^{-\beta }}{\alpha^{2}-\alpha^{-1}},f\left(\alpha =1\right)=1$$

where *σ_M_* is reduced stress, *σ* is nominal stress, *G_c_* is elastic modulus due to chemical crosslink and trapped entanglements, *G_e_* is the modulus contributed by the topological tube-like constraints or entanglements, *β* is an empirical parameter describing the relation between a deformed tube in a stretched state and an undeformed tube corresponding to an equilibrium state, and the value is taken as 1. α is the ratio of *l* (the stretched length of the rubber sample) to *l*_0_ (the original length of the rubber sample) during uniaxial deformation.

**Figure 6:**
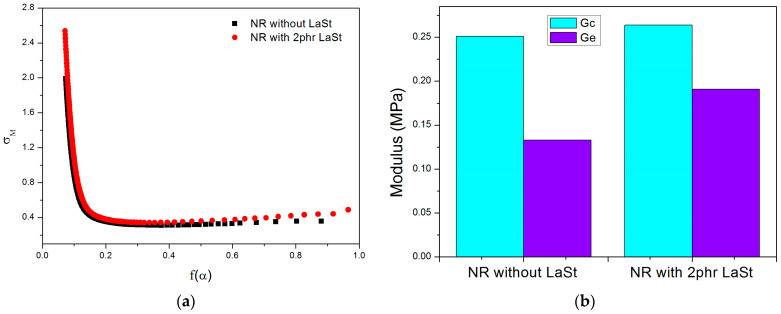
The Mooney–Rivlin plots of reduced stress (**a**) and parameters G_c_ and G_e_ (**b**) of SCR-WF without LaSt and 2 phr LaSt.

The corresponding Mooney–Rivlin plots are shown in Figure 6a, and the fitting parameters are given in Figure 6b. An obvious stress reduction (σ_M_) is observed at low strains, which is due to the relaxation or slippage of the natural rubber molecular chains leading to entanglement. Subsequently, the decreasing stress gradually increases with increasing strain due to different strain-induced crystallization behaviors during stretching [45].

Both Gc and Ge values are larger than that of SCR-WF with the addition of LaSt, indicating the contribution of LaSt in both crosslinking and topological constraints. From Table 2, it is obvious that the crosslink density of NR networks increases from 10.56 ± 0.17 × 10^−5^ mol·cm^−3^ to 12.90 ± 0.16 × 10^−5^ mol·cm^−3^ after the addition of 2 phr LaSt, indicating the formation of some new crosslinking points in the NR vulcanizates. A high Ge value means that the NR molecular chains are more entangled after adding LaSt. The polymer molecules can be easily absorbed on the surface of LaSt, forming the new complex due to the coordination between the La ion and O, N, and P atoms of protein and phospholipids in NR. Finally, the entanglement of bulk rubber with a new complex, developing new physical network crosslinking points, enhances the mechanical properties of NR.

**Figure 7:**
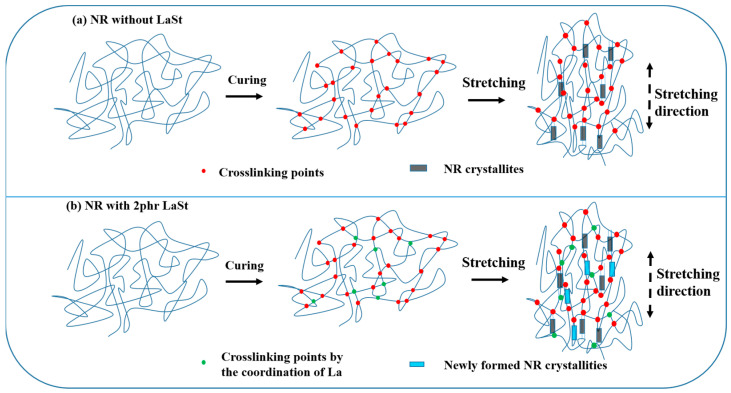
Schematic models of the role of LaSt on the SIC of vulcanized SCR-WF.

A schematic model of the role of LaSt on the SIC of vulcanized SCR-WF based on the obtained results can be proposed, as shown in Figure 7. During the cure process of NR, some new complex may be formed by the coordination between the La ion and O, N, and P atoms of protein and phospholipids, resulting in higher M_H_ and M_H_−M_L_. The swelling results demonstrate that the presence of LaSt significantly increases the crosslinking density of the vulcanized SCR-WF at lower LaSt loading. In addition, some “instantaneous complex” can be produced when the rubber vulcanizates are stretched, because macromolecules are forced to arrange quickly and distance between La atoms and the ligand narrows rapidly. Only under a large enough deformation will a growing number of crystals during stretching be formed. Due to the new complex and some “instantaneous complex”, the crystallinity index is higher than pure rubber, above the onset strain of SIC of SCR-WF with LaSt.

## 4. Conclusions

In this paper, we aimed to illuminate the role of LaSt on the SIC behavior and mechanical properties of SCR-WF. The addition of LaSt has taken the cure reaction, but it cannot accelerate the vulcanization of SCR-WF. Meanwhile, the addition of LaSt enhances the tensile strength, tear strength, and fracture toughness of NR vulcanizates. In addition, the crosslink densities of SCR-WF increase with increasing LaSt loading. Moreover, the addition of LaSt cannot accelerate the onset strain of SIC of SCR-WF, although the crosslink density of vulcanized SCR-WF with LaSt is higher than pure rubber vulcanizates. Only when the strain is larger than 3.5 does the vulcanized SCR-WF with 2 phr LaSt exhibit a higher crystallinity index and value of <$P_{2}^{am}$> than SCR-WF without LaSt, which indicates that LaSt can enhance the crystallinity index. The tube model further indicates the contribution of LaSt in both crosslinking and topological constraints. To summarize, the work acquires a better understanding of the role of LaSt on the SIC behavior and mechanical properties of SCR-WF. The research results of this paper provide theoretical guidance for the preparation of SCR-WF with superior mechanical properties and help expand the application of SCR-WF in rubber products.

## Figures and Tables

**Table 1:** Cure characteristics of NR compound with different LaSt loadings.

LaSt Loading	0	0.5	1.0	1.5	2
M_L_ (dNm)	0.801	0.409	0.451	0.461	0.515
M_H_ (dNm)	4.706	4.741	5.242	5.297	5.299
M_H_ − M_L_ (dNm)	3.905	4.332	4.791	4.836	4.784
T_S1_ (min)	3.84	3.13	3.05	2.87	2.95
T_90_ (min)	22.03	21.67	21.77	22.07	21.97
CRI	5.49	5.39	5.34	5.20	5.25

**Table 2:** Effect of LaSt loading on the crosslink densities of NR vulcanizates.

LaSt Loading	0	0.5	1.0	1.5	2
Swelling rate	5.73 ± 0.04	5.57 ± 0.01	5.54 ± 0.04	5.38 ± 0.01	5.25 ± 0.03
Crosslink density (×10^−5^ mol·cm^−3^)	10.56 ± 0.17	11.25 ± 0.05	11.44 ± 0.18	12.18 ± 0.06	12.90 ± 0.16

**Table 3:** Dependence of mechanical properties of SCR-WF with different LaSt contents.

LaSt Loading	0	0.5	1.0	1.5	2
Tensile strength (MPa)	18.12 ± 0.85	18.98 ± 1.18	20.61 ± 1.04	22.78 ± 1.04	23.01 ± 0.36
Elongation at break (%)	813 ± 20	805 ± 14	797 ± 14	816 ± 16	809 ± 25
Modulus (100%) (MPa)	0.51 ± 0.03	0.52 ± 0.02	0.55 ± 0.03	0.58 ± 0.03	0.59 ± 0.04
Modulus (300%) (MPa)	1.24 ± 0.04	1.24 ± 0.01	1.33 ± 0.03	1.38 ± 0.04	1.40 ± 0.03
Modulus (500%) (MPa)	2.84 ± 0.17	2.97 ± 0.03	3.32 ± 0.12	3.39 ± 0.21	3.44 ± 0.24

## Data Availability

Data are contained within the article.
